# Childhood trauma and suicidal ideation in female prisoners: serial mediation via intolerance of uncertainty and emotional problems

**DOI:** 10.3389/fpsyg.2026.1935504

**Published:** 2026-10-02

**Authors:** Kun-yan Wang, Jue Deng, Xiang-hui Lai, Ruo-Heng Wang, Xiao-Li Zhu

**Affiliations:** 1 Department of Penalty Execution, Fujian Police College, Fuzhou, China; 2 Narcotics Control Research Center of Southeastern China, Fujian Police College, Fuzhou, China; 3 Department of Basic Courses, Fujian Police College, Fuzhou, China

**Keywords:** childhood trauma, suicidal ideation, intolerance of uncertainty, emotional problems, female prisoners, serial mediation

## Abstract

**Introduction:**

Given the high risk of suicide among female prisoners, investigating the internal risk factors and pathways associated with suicidal ideation holds substantial clinical and practical significance. Childhood trauma, a critical distal risk factor, is closely associated with suicidal behavior. However, the specific cognitive and affective mechanisms through which childhood trauma links to suicidal ideation remain underexplored. Drawing on Emotion Dysregulation Theory and related research, this study constructed a serial mediation model to explore the relationship between childhood trauma and suicidal ideation in female prisoners, as well as the mediating roles of intolerance of uncertainty and emotional problems.

**Methods:**

A cross-sectional survey using standardized questionnaires was conducted with a sample of 1,086 female prisoners.

**Results:**

The results indicated that childhood trauma showed a significant indirect association with suicidal ideation through intolerance of uncertainty and emotional problems. Specifically, emotional problems showed a significant independent mediating effect, and intolerance of uncertainty was involved in a significant serial mediation pathway linking childhood trauma to suicidal ideation. Furthermore, the direct paths from childhood trauma and intolerance of uncertainty to concurrent suicidal ideation were not statistically significant. Both variables were positively associated with emotional problems, which were associated with higher levels of suicidal ideation.

**Discussion:**

This study delineates the cognitive-affective pathways through which early trauma is indirectly associated with suicidal ideation, providing empirical evidence for targeted suicide risk assessment and psychological interventions in correctional settings.

## 1 Introduction

Research on the mental health of incarcerated individuals is not only a crucial component of risk assessment but also a shared priority in both the judicial and public health sectors. Previous studies have established that suicide is among the leading causes of death in correctional environments. According to a recent systematic review (Mundt et al., 2024), the incidence of suicide among female prisoners is approximately 10.4 to 32.4 times higher than that of the general population. This disproportionately high risk may be closely linked to the unique physiological characteristics, social roles, and traumatic experiences of female inmates. Compared to their male counterparts, female prisoners exhibit a significantly higher prevalence of mental health issues, including post-traumatic stress disorder (PTSD), depression, and anxiety. Notably, childhood trauma has been identified as a core predictor of suicide risk among female inmates (Clements-Nolle et al., 2009).

Adverse childhood experiences (ACEs) are associated with profound and enduring mental health consequences. Research funded by the U. S. Centers for Disease Control and Prevention (CDC) indicates that exposure to childhood trauma accounts for an estimated 67% of the population-attributable risk for lifetime suicide attempts (Dube et al., 2001). Furthermore, an empirical comorbidity survey reported that experiences of childhood physical abuse, sexual abuse, or witnessing domestic violence are associated with suicidal ideation in 16% of women and suicide attempts in 50% of women (Roy, 2012). Within correctional settings, up to 76.3% of female inmates report experiencing various forms of childhood trauma. This prevalence is significantly higher than that observed in both male incarcerated populations (Ford et al., 2015) and the general public (where rates of childhood physical and sexual abuse among women are approximately 20 and 13%, respectively; Dye and Aday, 2013; Roy, 2012; Annett et al., 2023). Concurrently, the female prison environment presents unique stressors, such as guilt stemming from maternal deprivation, the loss of family roles, and the severance of intimate relationships during incarceration. These environmental factors may be relevant to the association between childhood trauma and current mental health difficulties.

### 1.1 Childhood trauma and suicidal ideation

Childhood trauma typically encompasses various adverse experiences, such as emotional, physical, and sexual abuse, as well as neglect. Empirical studies indicate that the prevalence of childhood physical and sexual abuse among female inmates is significantly higher than that in the general population (Ford et al., 2007). Extensive epidemiological evidence further confirms that childhood trauma is a critical distal risk factor for suicidal behavior, with the cumulative number of traumatic events demonstrating a significant dose–response relationship with suicide risk (Baldini et al., 2025; Dube et al., 2001). For instance, previous surveys reveal that individuals who experienced childhood sexual abuse can face a suicide risk several to tens of times higher than that of the general population (Joiner et al., 2005). A recent meta-analysis further confirmed that, specifically among incarcerated populations, childhood sexual abuse, physical abuse, emotional abuse, and physical neglect are all significantly associated with increased suicide attempts (Nabinger et al., 2024).

Childhood adversity is robustly associated with long-term deleterious outcomes across neurobiological and psychosocial domains (Turecki and Brent, 2016). Such early trauma exposure is associated with poorer mental health and may also be indirectly associated with suicide risk through its links with emotion regulation and executive control (Teicher and Samson, 2016).

This long-term latent effect of childhood trauma may be linked to suicidal behavior through a dual pathway of cognition and emotion. Cognitively, childhood trauma is associated with lower tolerance for uncertainty and greater susceptibility to anxiety and helplessness under external stressors. Affectively, early traumatic experiences are closely linked to persistent difficulties in emotion regulation, clinically manifesting as exacerbated depressive and anxious symptoms, alongside significantly heightened irritability (Bernstein et al., 2003). Emotional dysregulation, in turn, is a proximal correlate of suicidal ideation.

Nevertheless, existing literature focusing on incarcerated populations remains subject to certain limitations. Most studies primarily focus on the direct main effects of childhood trauma on suicide risk, leaving the underlying indirect mechanisms and complex pathways underexplored. Particularly among female inmates—a population with unique psychosocial characteristics—the indirect associations linking childhood trauma, cognitive-affective factors, and suicidal ideation remain insufficiently examined.

### 1.2 The mediating role of intolerance of uncertainty

In recent years, Intolerance of Uncertainty (IU) has garnered extensive attention in clinical settings as a transdiagnostic psychopathological factor. IU reflects a trait-like tendency to exhibit negative cognitive, emotional, and behavioral responses to unknown or ambiguous situations (Carleton, 2012). As a higher-order vulnerability mechanism, elevated IU plays a critical role in anxiety, depressive, and impulse-control disorders. It is associated with heightened emotional dysregulation, which in turn shows associations with suicidal ideation and behavior (McEvoy and Mahoney, 2012). Regarding its role in bridging childhood trauma and suicide risk, Martin et al. (2021) demonstrated that IU partially mediates the relationship between childhood trauma and suicidal ideation. More recently, Jones et al. (2024) confirmed this mediating pathway in a sample of adolescents and young adults, showing that IU significantly mediates the association between childhood adversity and suicide risk. Furthermore, this indirect pathway may vary depending on gender differences and environmental stressors.

For female inmates, the information-deprived nature of the prison environment (e.g., delayed access to external information) and future uncertainties (e.g., ambiguous resocialization prospects and unstable intimate relationships) may be associated with greater deleterious effects of low tolerance for uncertainty (i.e., high IU). Neurobiological evidence indicates that, compared to men, women frequently exhibit amygdala hyperactivation when appraising threats in ambiguous situations, which is related to heightened subjective emotional arousal and stress reactivity (Domes et al., 2010). Within the correctional environment, a perceived loss of control (e.g., difficulties adapting to changing prison regulations or concerns about external social changes) may be associated with beliefs that “the world is unpredictable” and with difficulties tolerating uncertainty.

### 1.3 The mediating role of emotional problems

Emotional problems—such as core symptoms of depression, anxiety, and irritability—play a crucial mediating role between childhood trauma and suicidal ideation. According to Emotion Dysregulation Theory, childhood trauma is associated with hyperreactivity in the limbic system, manifesting as a significant impairment in emotion regulation capabilities (Gratz and Roemer, 2004). In correctional settings, specific stressors (e.g., social isolation and deprivation of control) are associated with stronger negative emotions, a pattern that may link trauma, emotional dysregulation, and suicide risk. Neurobiological research further indicates that childhood trauma is associated with disruptions in neural circuits underlying emotion regulation (e.g., weaker functional connectivity between the amygdala and the prefrontal cortex) and with exaggerated emotional responses to stress (McLaughlin et al., 2015). Additionally, clinical studies on patients with Borderline Personality Disorder (BPD) indicate that chronic emotional instability and impulsive reactivity are associated with early childhood trauma and with suicidal ideation (Crowell et al., 2009).

Female inmates face a dual burden of highly gender-specific environmental stressors: maternal deprivation and relational bullying. Research shows that maternal deprivation is directly associated with a profound and prolonged accumulation of guilt and perceived burdensomeness (Celinska and Siegel, 2010). Meanwhile, the prevalence of relational bullying remains persistently high within female correctional facilities. These prison stressors may be relevant to the associations between emotional symptoms—such as depression, anxiety, and irritability—and suicidal ideation among female inmates.

### 1.4 Present study

Given the elevated suicide risk among female inmates, examining the psychological factors associated with early trauma and current suicidal ideation is of critical clinical importance. Emotion Dysregulation Theory provides a framework for considering associations among early traumatic experiences, current cognitive-affective factors, and suicidal ideation. While the individual roles of intolerance of uncertainty (IU) and emotional problems have been documented, their potential indirect associations linking childhood trauma and suicidal ideation within correctional settings require systematic investigation. Exploring this cognitive-affective pathway can inform proactive risk assessment and targeted psychological interventions for female inmates. Therefore, this study constructs a serial mediation model to investigate the association between childhood trauma and suicidal ideation among female inmates, specifically examining the mediating roles of IU and emotional problems (see Figure 1). The specific research hypotheses are as follows:

**Figure 1:**
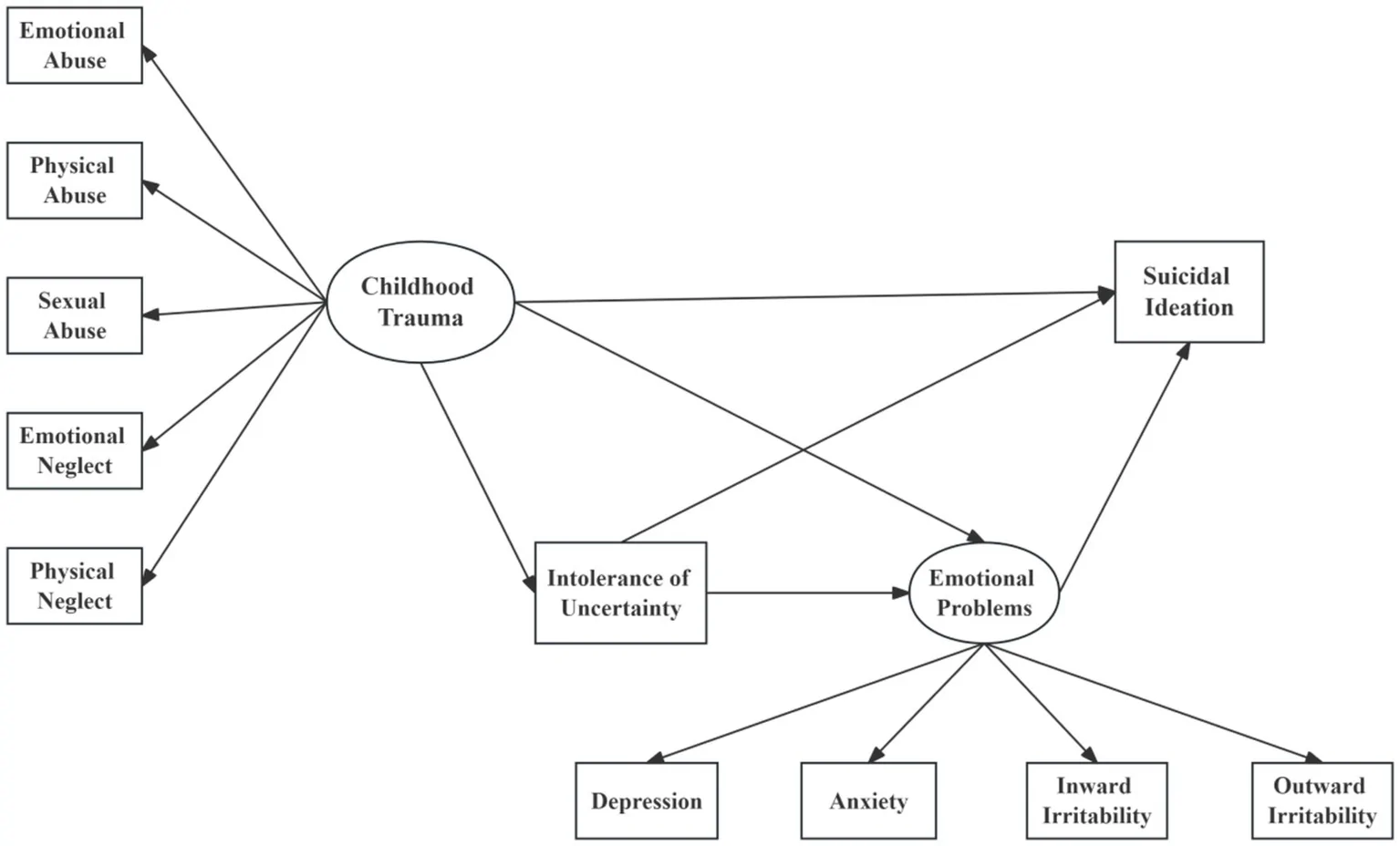
The hypothesized model.

*Hypothesis* 1 (H1): Childhood trauma is positively associated with suicidal ideation among female inmates.

*Hypothesis* 2 (H2): IU mediates the relationship between childhood trauma and suicidal ideation.

*Hypothesis* 3 (H3): Emotional problems mediate the relationship between childhood trauma and suicidal ideation.

*Hypothesis* 4 (H4): IU and emotional problems exert a serial mediating effect between childhood trauma and suicidal ideation.

## 2 Materials and methods

### 2.1 Participants

Data were gathered from female offenders recruited from a women‘s prison in a southeastern province of China. A total of 1,264 offenders were invited to complete several demographic questions and questionnaires measuring childhood trauma, intolerance of uncertainty, emotional problems, and suicidal ideation. The inclusion criteria were as follows: (1) willing to participate voluntarily and to provide written informed consent; (2) sufficient cognitive abilities to complete the self-report questionnaires; (3) aged 18 years or older; and (4) not under the influence of any substance at the time of participation. Those considered by prison officers to be too emotionally or physically frail to participate, or those with severe mental disorders (e.g., schizophrenia, acute manic episode) that precluded valid self-report, were excluded. No detainees or suspects were included in our study as they are held in other transitional facilities or on remand; all participants in this study were convicted prisoners with determined sentence lengths.

Data collection was conducted in August 2023. All recruitment and survey procedures were carried out independently by the research team; correctional officers assisted only with security and logistical arrangements and did not participate in the informed consent process or questionnaire administration. Prior to enrollment, participants were informed that their participation was entirely voluntary, that all responses would be anonymous and confidential, that no individual data would be accessible to prison staff, and that neither participation nor refusal would affect evaluations of their conduct in prison, parole eligibility, or treatment in day-to-day prison management. They were also told that they could withdraw at any time without providing a reason and without any negative consequences. Written informed consent was obtained from all participants before they completed the paper-and-pencil questionnaires independently in a quiet classroom setting. During the informed consent process, participants were informed that although all data were anonymous and confidential, the research team would take immediate action if a participant showed signs of acute distress during the survey, including offering on-site support and referral to the prison’s psychological counseling unit if needed.

After a screening process, the data of 178 participants were discarded due to patterned responding (defined as selecting the same response option for ≥7 consecutive items), missing data exceeding 10%, or elevated scores on the three CTQ validity items (items 10, 16, and 26; total score ≥12). For retained cases, item-level missing values were handled using person mean imputation. Therefore, data from 1,086 participants were included in the final analysis, yielding an effective response rate of 85.9%. The sample size substantially exceeded the minimum requirement for structural equation modeling (Bentler and Chou, 1987), with a case-to-parameter ratio exceeding 10:1.

The mean age of the participants was 36.84 years (SD = 9.18). Regarding educational attainment, 685 (63.1%) had completed middle school or below, 205 (18.9%) had completed high school or vocational school, 175 (16.1%) held a college degree or higher, and 21 (1.9%) did not report their education level. Marital status was distributed as follows: 333 (30.7%) were unmarried, 450 (41.4%) were married (including first marriages and remarriages), 270 (24.9%) were divorced, 12 (1.1%) were widowed, and 21 (1.9%) did not disclose their marital status.

This study was approved by the Institutional Review Board of the first author’s affiliated institution (Approval No. EC-20230824-1022), and written informed consent was obtained from all participants.

### 2.2 Measures

#### 2.2.1 Suicidal ideation scale

Suicidal ideation, the outcome variable of this study, was measured using the Beck Scale for Suicide Ideation (BSI; Beck et al., 1997). Originally developed as the first clinical tool for assessing suicidal ideation, the BSI was later translated and revised by the Beijing Suicide Research and Prevention Center in 2010 to evaluate the intensity of suicidal ideation and suicide risk in Chinese populations (Li et al., 2010). The Chinese version of the BSI consists of 19 items and comprises two subscales: a 5-item suicidal ideation screening subscale (items 1–5) and a 14-item suicidal tendency subscale (items 6–19). Given that the outcome variable of this study is the intensity of suicidal ideation rather than the detailed behavioral components of suicide risk, we utilized only the five-item screening subscale, a practice consistent with prior research (Li et al., 2010). Items are rated on a 3-point Likert scale ranging from 0 to 2, with higher total scores indicating stronger suicidal ideation. In the current sample of female inmates, the Chinese version of the BSI demonstrated excellent internal consistency (McDonald’s Ω = 0.882, Cronbach’s α = 0.873).

#### 2.2.2 Childhood trauma questionnaire

Childhood trauma, the primary predictor variable, was assessed using the Childhood Trauma Questionnaire (CTQ; Bernstein et al., 2003). The Chinese version of the CTQ, revised by Fu et al. (2005), has demonstrated good reliability and validity. It comprises 28 items (25 clinical items and three validity items) rated on a 5-point Likert scale. The questionnaire assesses five factors: emotional abuse, physical abuse, sexual abuse, emotional neglect, and physical neglect. In this study, the overall Chinese version of the CTQ showed acceptable internal consistency (McDonald’s Ω = 0.747, Cronbach’s α = 0.703). The reliability coefficients for the five specific subscales were as follows: emotional abuse (Ω = 0.718, α = 0.707), physical abuse (Ω = 0.823, α = 0.808), sexual abuse (Ω = 0.813, α = 0.804), emotional neglect (Ω = 0.787, α = 0.781), and physical neglect (Ω = 0.714, α = 0.698).

#### 2.2.3 Intolerance of uncertainty scale

Intolerance of uncertainty, the first mediating variable, was measured using the Intolerance of Uncertainty Scale-12 (IUS-12). Originally developed by Carleton et al. (2007), the scale was translated into Chinese and validated by Wu et al. (2016) and Zhang et al. (2017). It comprises 12 items rated on a 5-point Likert scale, ranging from 1 (completely disagree) to 5 (completely agree), with higher scores indicating a lower tolerance for uncertainty. In the current sample, the Chinese version of the IUS-12 demonstrated excellent internal consistency (McDonald’s Ω = 0.852, Cronbach’s α = 0.851).

#### 2.2.4 Irritability, depression, and anxiety scale

Emotional problems, the second mediating variable, were assessed using the Irritability, Depression, and Anxiety Scale (IDA). Initially developed by Snaith et al. (1978), the IDA was translated into Chinese and validated by Yuan et al. (2002). The scale consists of 18 symptom-based items rated on a 4-point Likert scale (0–3) and measures four factors: depression, anxiety, inward irritability, and outward irritability. In this study, the overall Chinese IDA exhibited good internal consistency (McDonald’s Ω = 0.837, Cronbach’s α = 0.832). The reliability coefficients for the four subscales were as follows: depression (Ω = 0.747, α = 0.628), anxiety (Ω = 0.822, α = 0.703), inward irritability (Ω = 0.730, α = 0.730), and outward irritability (Ω = 0.635, α = 0.622). Although the alpha values for depression and outward irritability fell in the lower acceptable range, this pattern is not uncommon for brief subscales with few items and for samples drawn from correctional populations, where response variability may be constrained (Yuan et al., 2002). Given that the overall scale reliability remained satisfactory and all subscales reflect clinically relevant dimensions, the composite scores were retained for analysis.

### 2.3 Data analysis

Statistical analyses were conducted using SPSS 26.0 and Python (version 3.9). Structural equation modeling (SEM) was performed with maximum likelihood (ML) estimation, which is appropriate for continuous indicator scores. Given that several indicators departed from normality, Spearman’s rho was adopted for the descriptive correlations, and statistical inference in the model relied on bias-corrected bootstrap confidence intervals, which do not depend on normality assumptions. In the model, childhood trauma was specified as a latent variable indicated by the five subscale scores of the Childhood Trauma Questionnaire (emotional abuse, physical abuse, sexual abuse, emotional neglect, and physical neglect), and emotional problems were specified as a latent variable indicated by the four subscale scores of the Irritability, Depression, and Anxiety Scale (depression, anxiety, inward irritability, and outward irritability); intolerance of uncertainty and suicidal ideation were included as observed variables (total scores). Four residual covariances were specified between conceptually overlapping dimensions: depression with anxiety, inward with outward irritability, emotional with physical neglect, and anxiety with inward irritability. The model controlled for marital status and drug use history. Item-level missing values on the multi-item scales were handled using person mean imputation, whereas missing values on the covariates were handled using full-information maximum likelihood (FIML) estimation, which retains all available cases. Model fit was evaluated against the following criteria: χ^2^/df < 5, RMSEA < 0.08, CFI > 0.90, and TLI > 0.90. Serial mediation effects were tested using bias-corrected bootstrap confidence intervals with 5,000 resamples; an effect was considered statistically significant if the 95% confidence interval (CI) did not include zero. The significance level was set at α = 0.05.

Common method bias was assessed using Harman’s single-factor test. Principal component analysis revealed 14 factors with eigenvalues greater than 1. The first factor accounted for 18.5% of the total variance, which is well below the 40% critical threshold, indicating that common method bias was not a significant concern in this study.

## 3 Results

### 3.1 Descriptive statistics and correlation analyses

The means (M), standard deviations (SD), and Spearman’s correlation coefficients of the main variables are presented in Table 1. The results showed that childhood trauma was significantly and positively correlated with intolerance of uncertainty (*p* < 0.01), emotional problems (*p* < 0.01), and suicidal ideation (*p* < 0.01). Furthermore, intolerance of uncertainty exhibited significant positive correlations with both emotional problems (*p* < 0.01) and suicidal ideation (*p* < 0.01). Emotional problems were also positively associated with suicidal ideation (*p* < 0.01). Additionally, among the sub-dimensions of childhood trauma, all abuse dimensions (emotional, physical, and sexual) were positively correlated with intolerance of uncertainty (*p* < 0.01), whereas the neglect dimensions (emotional and physical) showed no significant correlations. These findings provided preliminary support for testing the proposed serial mediation model. Based on theoretical considerations, marital status and drug use history were included as covariates in the subsequent structural equation model (Celinska and Siegel, 2010; Zheng et al., 2021).

**Table 1:** Descriptive statistics and correlation matrix for each variable (*N* = 1,086).

Variable	M	SD	1	2	3	4	5	6	7	8	9	10	11	12
Childhood trauma^1^	36.66	9.91												
Emotional abuse^2^	6.66	2.63	0.552^**^											
Physical abuse^3^	5.58	1.88	0.463^**^	0.370^**^										
Sexual abuse^4^	5.47	1.55	0.305^**^	0.257^**^	0.181^**^									
Emotional neglect^5^	10.19	4.48	0.853^**^	0.339^**^	0.314^**^	0.148**								
Physical neglect^6^	8.76	2.88	0.787^**^	0.252^**^	0.318^**^	0.174**	0.521^**^							
Intolerance of uncertainty^7^	25.47	8.08	0.128^**^	0.290^**^	0.177^**^	0.138**	0.046	0.056						
Emotional problems^8^	17.88	7.52	0.333^**^	0.359^**^	0.222^**^	0.150**	0.250^**^	0.220^**^	0.395^**^					
Depression^9^	5.07	2.51	0.251^**^	0.204^**^	0.132^**^	0.104**	0.218^**^	0.150^**^	0.259^**^	0.756^**^				
Anxiety^10^	6.11	2.48	0.252^**^	0.270^**^	0.165^**^	0.107**	0.172^**^	0.188^**^	0.351^**^	0.786^**^	0.526^**^			
Inward irritability^11^	3.37	2.57	0.293^**^	0.356^**^	0.218^**^	0.137**	0.204^**^	0.199^**^	0.378^**^	0.837^**^	0.477^**^	0.524^**^		
Outward irritability^12^	3.34	2.04	0.287^**^	0.303^**^	0.182^**^	0.134**	0.210^**^	0.196^**^	0.241^**^	0.678^**^	0.295^**^	0.353^**^	0.600^**^	
Suicidal ideation	0.83	1.80	0.279^**^	0.325^**^	0.235^**^	0.114**	0.215^**^	0.182^**^	0.260^**^	0.481^**^	0.381^**^	0.304^**^	0.488^**^	0.312^**^

M, mean; SD, standard deviation. *p < 0.05, **p < 0.01, ***p < 0.001. Superscript numbers identify the variables corresponding to columns 1–12: 1 = childhood trauma; 2 = emotional abuse; 3 = physical abuse; 4 = sexual abuse; 5 = emotional neglect; 6 = physical neglect; 7 = intolerance of uncertainty; 8 = emotional problems; 9 = depression; 10 = anxiety; 11 = inward irritability; 12 = outward irritability.

### 3.2 Model fit and indirect effect analyses

A structural equation model (SEM) was employed to examine the serial mediation effect, with childhood trauma as the predictor variable, intolerance of uncertainty and emotional problems as mediating variables, and suicidal ideation as the outcome variable. The results indicated an acceptable model fit (*χ*^2^/*df* = 4.57, RMSEA = 0.060, CFI = 0.939, TLI = 0.902). Figure 2 illustrates the standardized path coefficients and their significance within the serial mediation model, while Table 2 presents the effect estimates and confidence intervals for both direct and indirect paths.

**Figure 2:**
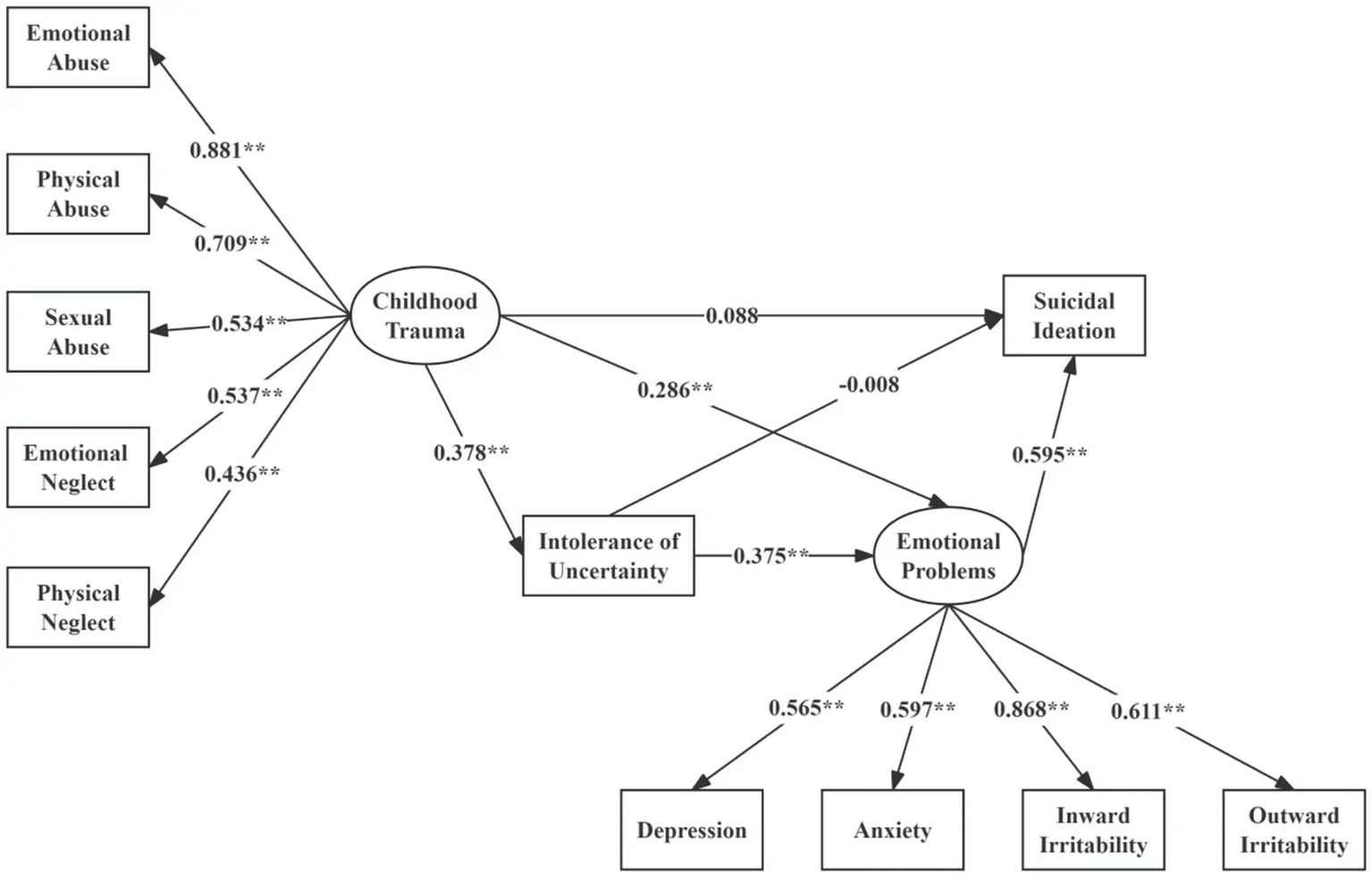
Serial mediation model of childhood trauma, intolerance of uncertainty, emotional problems, and suicidal ideation in female inmates (*N* = 1,086). Coefficients are standardized estimates. The model controlled for marital status and drug use history.

**Table 2:** Direct and indirect effects among childhood trauma, intolerance of uncertainty, emotional problems, and suicidal ideation in female inmates (*N* = 1,086).

Path	β	*SE*	95% CI		*p*
			Lower	Upper	
Direct path					
Childhood trauma → Suicidal ideation	0.088	0.057	−0.024	0.207	0.122
Indirect paths					
Childhood trauma → Intolerance of uncertainty → Suicidal ideation	−0.004	0.015	−0.035	0.026	0.818
Childhood trauma → Emotional problems → Suicidal ideation	0.171	0.036	0.099	0.244	<0.001
Childhood trauma → Intolerance of uncertainty → Emotional problems → Suicidal ideation	0.085	0.015	0.057	0.116	<0.001

Estimate = standardized β coefficient. SE, 95% CI, and *p* values are based on 5,000 bootstrap resamples. Marital status and drug use history were included as covariates.

Specifically, the findings revealed the following: (1) among female inmates, the direct path from childhood trauma to concurrent suicidal ideation was not statistically significant (*β* = 0.088, *p* = 0.122); significant indirect associations were observed through the specified mediation pathways. (2) Childhood trauma was significantly and positively associated with intolerance of uncertainty (*β* = 0.378, *p* < 0.01). Intolerance of uncertainty was positively associated with emotional problems, including depression, anxiety, and inward and outward irritability (*β* = 0.375, *p* < 0.01). This serial mediation path was statistically significant (Indirect Effect = 0.085, 95% CI [0.057, 0.116], *p* < 0.001). (3) Additionally, childhood trauma showed a positive direct association with emotional problems (*β* = 0.286, *p* < 0.01), which were associated with stronger suicidal ideation (*β* = 0.595, *p* < 0.01). This indirect path was also significant (Indirect Effect = 0.171, 95% CI [0.099, 0.244], *p* < 0.001). (4) Regarding the hypotheses, the direct path from childhood trauma to suicidal ideation was not statistically significant, leaving Hypothesis 1 unsupported. Intolerance of uncertainty did not exhibit a significant independent mediating effect, indicating that Hypothesis 2 was not supported. Emotional problems exerted a significant independent mediating effect, supporting Hypothesis 3. Intolerance of uncertainty and emotional problems formed a significant serial mediation pathway linking childhood trauma to suicidal ideation, providing support for Hypothesis 4. These conclusions remained unchanged after controlling for the covariates.

## 4 Discussion

This study constructed and tested a serial mediation model using a sample of female inmates to systematically explore the independent and serial mediating roles of intolerance of uncertainty and emotional problems in the relationship between childhood trauma and suicidal ideation. The findings describe cognitive and emotional factors involved in the association between childhood trauma and suicidal ideation among female inmates. Furthermore, these results offer empirical evidence for mental health education and crisis interventions tailored to female inmates. They may also inform future evaluations of psychological support strategies addressing emotional problems and suicidal ideation within correctional environments.

### 4.1 The relationship between childhood trauma and suicidal ideation

This study found that the total childhood trauma score and its sub-dimensions were significantly and positively correlated with suicidal ideation among female inmates. This indicates that those who have experienced more childhood adversity tend to exhibit higher levels of suicidal ideation. This finding is highly consistent with previous epidemiological studies on adverse childhood experiences (ACEs) and the mental health of female inmates (Mundt et al., 2024). The observed association adds to research on childhood trauma and suicidal ideation in this population (Zheng et al., 2021).

As a unique population, female inmates face highly complex and specific stressors within the prison environment. Beyond adapting to strict prison rules and information-deprived environments, they also endure guilt from maternal deprivation, the loss of family roles, and the severing of intimate relationships (Douglas et al., 2009). These distinct real-world hardships may be relevant to the psychological burden associated with early adverse experiences. Regarding specific trauma types, our results revealed that emotional abuse had the strongest association with suicidal ideation. Emotional abuse—such as persistent belittlement, neglect, and emotional deprivation—is associated with lower self-worth. This may contribute to negative core beliefs of being “unlovable” and reduced perceived safety in interpersonal relationships. Meanwhile, although emotional and physical neglect showed relatively weaker associations with suicidal ideation, these correlations remained statistically significant. This suggests that less visible forms of trauma, such as chronically unmet emotional or physical needs, are also relevant to concurrent suicidal ideation. They are often indirectly associated with increased suicide risk through links with chronic loneliness, depleted social support systems, or undermined physiological health foundations (Zheng et al., 2021).

Notably, while correlation analyses revealed a tight link between childhood trauma and suicidal ideation, the subsequent structural equation model showed that the direct path from childhood trauma to concurrent suicidal ideation was not statistically significant. This finding further clarifies the boundary conditions of childhood trauma: as an early life event, it does not directly equate to current suicidal ideation. Instead, it lies dormant within the individual as an underlying “psychological vulnerability.” Female inmates with severe childhood adversity may report lower internal psychological capital and perceived security when facing incarceration stressors. This distal trauma history may be linked to impaired cognitive frameworks and emotion regulation, which are in turn associated with concurrent suicidal ideation.

### 4.2 The mediating role of intolerance of uncertainty

Structural equation modeling showed that childhood trauma was positively associated with IU and that IU was positively associated with emotional problems. Specifically, higher levels of childhood trauma were significantly associated with lower tolerance for uncertainty among female inmates. Lower tolerance for uncertainty was significantly associated with greater emotional problems, including depression and anxiety. This finding is consistent with the conceptualization of IU as a cognitive vulnerability that, in the context of early adversity, may predispose individuals to heightened emotional reactivity (Carleton, 2016). For individuals who have experienced early adversity, their foundational psychological structures and sense of security are often compromised. Consequently, when facing ambiguous and unpredictable situations, they struggle to tolerate uncertainty and tend to exhibit hypervigilance and catastrophic evaluation (Hayward et al., 2020; Morriss et al., 2025).

Notably, this mediation mechanism exhibits high situational and gender specificity among female inmates. During socialization, women are typically reinforced to adopt a “relation-oriented” cognitive mode. Thus, establishing their sense of security in external environments relies more heavily on interpersonal stability and emotional support (Tamres et al., 2002). However, in the unique, gender-segregated environment of a prison, female inmates are severely deprived of their original external social support systems, such as family connections and intimate relationships (Douglas et al., 2009). The absence of an external interpersonal safety net is related to a heightened fear of losing control. Limited external support may be relevant to difficulties tolerating uncertainty in this setting. When individuals report low tolerance for prolonged ambiguity and loneliness, a pattern linking trauma experience, cognitive rigidity, and emotional distress may be observed (Zheng et al., 2021).

### 4.3 The mediating role of emotional problems

The model supported a significant indirect association between childhood trauma and suicidal ideation through emotional problems among female inmates. This pathway describes an association with concurrent suicidal ideation. According to Emotion Dysregulation Theory, childhood trauma is associated with disruptions in neuromodulatory circuits between the limbic system and the prefrontal cortex (Gross, 2015). These disruptions are associated with heightened emotional reactivity and sustained dysregulation in response to stress, and with lower psychological resilience. In the present model, emotional problems statistically linked childhood trauma with concurrent suicidal ideation.

The incarceration environment provides a relevant context for interpreting this indirect association among female inmates. Previous research indicates that social isolation, maternal deprivation, and relational bullying within prisons constitute distinct stressors for this population (Douglas et al., 2009). These factors are related to the depletion of emotion regulation resources and are closely associated with the continuous accumulation of depressive, anxious, and irritable symptoms. When individuals experience high levels of negative emotions for prolonged periods, their thwarted belongingness and perceived burdensomeness significantly intensify (Joiner, 2005). Consequently, suicidal ideation may emerge as a coping cognition aimed at “ending the pain.” Notably, the current study found a significant indirect pathway wherein intolerance of uncertainty is indirectly associated with suicidal ideation through its link with emotional problems. This further suggests that suicide risk among female inmates exhibits distinctly “emotion-driven” characteristics. Compared with cognitive biases, emotional problems—given their dynamic nature and sensitivity to environmental triggers (e.g., daily interpersonal conflicts)—may warrant particular attention as a proximal intervention target in efforts to address trauma-related suicidal ideation (Zheng et al., 2021).

### 4.4 The serial mediating effect of intolerance of uncertainty and emotional problems

The current study supports a serial indirect association in the specified model among female inmates: childhood trauma → intolerance of uncertainty → emotional problems → suicidal ideation. This pattern is consistent with a serial indirect association in which early negative life events are indirectly associated with suicidal ideation. The finding is also consistent with the Diathesis-Stress Model of suicidal behavior, which conceptualizes suicide risk as involving the interaction between internal vulnerabilities (e.g., personality traits, cognitive biases) and external stressful events (Abramson et al., 1989; Joiner, 2005). Specifically, childhood abuse and neglect are associated with a lower sense of control and reduced perceived predictability of the environment. Such difficulties may be associated with lower tolerance for uncertainty.

When facing the complex stressors of prison life, individuals with this cognitive bias may be more likely to catastrophize ambiguous information. This cognitive pattern is associated with severe anxiety, depression, and irritability, and with suicidal ideation, which has been described as an extreme means to “end the pain” (Hayward et al., 2020). Furthermore, the direct path from IU to suicidal ideation was not statistically significant in the specified model. Emotional problems were included in the significant serial indirect association, which is relevant to prior research on negative emotions and suicidal ideation (Zheng et al., 2021). Within this correctional sample, the analysis of cross-sectional data indicated a serial indirect association involving IU and emotional problems. Higher IU was associated with greater emotional problems, which were associated with higher concurrent suicidal ideation. This pattern suggests that cognitive restructuring and emotion regulation warrant further investigation as potential intervention approaches in correctional settings.

## 5 Implications and limitations

The findings of this study contribute to deepening the understanding of the factors associated with suicidal ideation among female prisoners and have practical implications for preventing and alleviating suicide risks in correctional facilities, as well as promoting targeted mental health management. First, intolerance of uncertainty (IU) represents a critical cognitive vulnerability and a potential target for future intervention research. Addressing female prisoners’ tolerance for uncertainty warrants consideration in research on correctional mental health education. Cognitive-behavioral interventions (Carleton, 2012), such as cognitive restructuring and uncertainty-focused exposure, may help support inmates’ psychological resources for coping with the unpredictable nature of the prison environment and their hypervigilance to trauma triggers. Addressing emotional problems warrants evaluation as a potential approach to reducing suicidal ideation. Given the observed indirect association through emotional problems, therapies targeting emotion regulation warrant further investigation. Group training based on Dialectical Behavior Therapy (DBT) or structured emotion regulation programs may help inmates learn to tolerate distress and manage severe anxiety and inward/outward irritability, potentially interrupting the pattern linking trauma-related distress and emotional problems. Third, the importance of rebuilding interpersonal connections and systemic social support within the justice education system should not be overlooked. Mental health practitioners in prisons can actively design collaborative psychological support initiatives—such as the Lilac Project—to facilitate holistic emotional rehabilitation and social reintegration. Furthermore, for inmates facing unique incarceration stressors such as maternal deprivation and relational bullying, integrating restorative justice and family support frameworks may help address depleted emotional resources and provide additional interpersonal support.

Despite revealing these underlying relationships, the current study has several limitations. First, the cross-sectional design precludes the establishment of strict causal relationships. Future research should employ longitudinal designs to examine the temporal relationships among IU, emotional problems, and suicidal ideation. Second, the core variables were assessed primarily through self-report questionnaires. Given the sensitive nature of trauma and suicide, the results may be subject to recall bias and social desirability effects. Future studies should incorporate multi-source data, such as evaluations by correctional officers or clinical interviews, for cross-validation. Finally, the sample was drawn from a single women’s prison in a southeastern province, limiting the generalizability of the findings due to regional differences in judicial culture. Future research should conduct cross-regional, multicenter collaborative investigations to further validate and broaden the generalizability of this model.

## 6 Conclusion

Based on a cross-sectional design, the results of this study support indirect associations consistent with a serial mediation model linking childhood trauma to suicidal ideation among female inmates. Notably, the structural equation model revealed a non-significant direct effect of childhood trauma on concurrent suicidal ideation. The non-significant direct path does not establish the absence of a direct association. Significant indirect associations were observed through emotional problems alone and through the specified serial pathway involving IU and emotional problems. Specifically, a history of childhood trauma is associated with a diminished tolerance for environmental uncertainty. This cognitive vulnerability is related to heightened emotional problems—such as depression, anxiety, and irritability—which are correlated with higher concurrent suicidal ideation. Furthermore, the direct path from IU to suicidal ideation was not statistically significant, whereas the serial indirect association involving IU and emotional problems was significant. These findings suggest that, in efforts to address suicide risk among female inmates, clinical interventions may need to look beyond trauma history and consider the cognitive and emotional factors identified in this model. Addressing both cognitive rigidity and emotional dysregulation may warrant further investigation as a potential intervention approach.

## Data Availability

The datasets generated during and/or analyzed during the current study are available from the corresponding author upon reasonable request. Due to the sensitive nature of data collected from incarcerated individuals and the confidential mental health information involved, access is subject to ethical and institutional restrictions and will be considered on a case-by-case basis in accordance with the approval of the institutional review board. Requests to access the datasets should be directed to Kun-yan Wang, psy19940323@163.com.
